# The utility of the respiratory rate-oxygenation index as a predictor of treatment response in dogs receiving high-flow nasal cannula oxygen therapy

**DOI:** 10.3389/fvets.2024.1404195

**Published:** 2024-05-07

**Authors:** Erin Duble, Jiwoong Her, Ingrid Preteseille, Jeongmin Lee, Bernard Allaouchiche, Céline Pouzot-Nevoret

**Affiliations:** ^1^Department of Veterinary Clinical Sciences, College of Veterinary Medicine, The Ohio State University, Columbus, OH, United States; ^2^Intensive Care Unit (SIAMU), VetAgro Sup, Université de Lyon, Marcy-l'Étoile, France; ^3^Department of Veterinary Clinical Sciences, College of Veterinary Medicine, Iowa State University, Ames, IA, United States; ^4^VetAgro Sup, Université de Lyon, Marcy-l'Étoile, France; ^5^Hospices Civils de Lyon, Service de Réanimation, Centre Hospitalier Lyon-Sud, Pierre-Bénite, France

**Keywords:** high-flow nasal cannula oxygen therapy, respiratory rate-oxygenation index, dogs, respiratory failure, mechanical ventilation

## Abstract

**Objective:**

This study aims to evaluate the respiratory rate-oxygenation index (ROX) and the ratio of pulse oximetry saturation (SpO_2_) to the fraction of inspired oxygen (FiO_2_) (SpO_2_/FiO_2_, [SF]) to determine whether these indices are predictive of outcome in dogs receiving high-flow nasal cannula oxygen therapy (HFNOT).

**Design:**

This is a prospective observational study.

**Setting:**

This study was carried out at two university teaching hospitals.

**Animals:**

In total, 88 dogs treated with HFNOT for hypoxemic respiratory failure due to various pulmonary diseases were selected.

**Measurements and main results:**

The ROX index was defined as the SF divided by the respiratory rate (RR). ROX and SF were calculated at baseline and for each hour of HFNOT. The overall success rate of HFNOT was 38% (*N* = 33/88). Variables predicting HFNOT success were determined using logistic regression, and the predictive power of each variable was assessed using the area under the receiver operating curve (AUC). ROX and SF were adequately predictive of HFNOT success when averaged over 0–16 h of treatment, with similar AUCs of 0.72 (95% confidence interval [CI] 0.60–0.83) and 0.77 (95% CI 0.66–0.87), respectively (*p* < 0.05). SF showed acceptable discriminatory power in predicting HFNOT outcome at 7 h, with an AUC of 0.77 (95% CI 0.61–0.93, *p* = 0.013), and the optimal cutoff for predicting HFNC failure at 7 h was SF ≤ 191 (sensitivity 83% and specificity 76%).

**Conclusion:**

These indices were easily obtained in dogs undergoing HFNOT. The results suggest that ROX and SF may have clinical utility in predicting the outcomes of dogs on HFNOT. Future studies are warranted to confirm these findings in a larger number of dogs in specific disease populations.

## Introduction

Hypoxemic respiratory failure (HRF) is a common diagnosis in patients presenting to emergency medical centers, both in human and veterinary medicine (1–3). There is an ever-growing interest in discovering means of oxygen delivery that maximize the efficiency and comfort of oxygen support while minimizing invasive instrumentation. High-flow nasal cannula oxygen therapy (HFNOT) has grown in popularity in human medicine as a method of bridging oxygen support between conventional oxygen therapy (COT) and mechanical ventilation (MV) (3, 4). It is useful in the management of HRF of many etiologies (2, 5–8). The benefits of HFNOT include the ability to provide 100% oxygen, the capacity to deliver positive end-expiratory pressure to increase alveolar recruitment, the provision of heated and humidified air to improve patient comfort, and the reduction of anatomical dead space (3, 9). More recently, HFNOT has become more widely utilized in dogs, and its efficacy in the management of canine HRF has been demonstrated in multiple retrospective and prospective studies (10–15). HFNOT is particularly appealing in veterinary medicine as it offers hope for a positive outcome in patients failing COT before owners have to consider the financial and emotional implications of MV.

With the increasing use of HFNOT in humans and dogs, the need for objective predictors of HFNOT response has come to light. Failure to identify inadequate response to HFNOT has been shown to delay timely escalation to MV and increase patient morbidity and mortality in several human studies (16). The respiratory-rate oxygenation index (ROX), which is defined as the ratio of oxygen saturation measured by pulse oximetry (SpO_2_) to the fraction of inspired oxygen (FiO_2_) (SF) divided by the respiratory rate (RR), has been validated as a predictive index of HFNOT outcome (1). Specific ROX cutoff values have been established to guide escalation from HFNOT to MV in humans (1, 4, 17). In addition, the modified ROX (ROX-HR), which is defined as ROX divided by the heart rate (HR) and then multiplied by a factor of 100, has also been shown to have good predictive utility in guiding escalation of HFNOT to MV (18, 19). Lower ROX, SF, and ROX-HR are associated with a higher likelihood of failure to respond to HNFT (1). The predictive utility of ROX, SF, and ROX-HR has been investigated in a recent retrospective study involving dogs with HRF treated with HFNOT (20). The results of this study support the use of ROX and SF when predicting the outcome of HFNOT in dogs, echoing findings from human studies. Specifically, a ROX value of ≤3.68 and an SF value of ≤143 at 6 h on HFNOT were found to be excellent predictive cutoffs for failure to respond to HFNOT.

The utility of ROX, SF, and ROX-HR in patients receiving HFNOT lies in their non-invasive nature and simplicity in point-of-care calculation. These indices operate independently of the primary diagnosis, relying on readily available objective variables, making them suitable for application in dogs on HFNOT. While these features make them appealing for application in dogs, to the best of the authors’ knowledge, there is only one existing retrospective study evaluating this application (20). The primary objective of this prospective study was to further validate the ability of ROX, SF, and ROX-HR to predict HFNOT outcomes in dogs with HRF. The secondary aim was to determine cutoff values for these indices at specific time points that could guide the decision to escalate oxygen support from HFNOT to MV in dogs. The authors hypothesized that these indices would be highly predictive of outcomes in dogs receiving HFNOT.

## Materials and methods

Prospective case enrollment occurred at two veterinary university teaching hospitals between August 2021 and January 2023. For the purposes of this investigation, HRF was defined as a PaO_2_ of <80 mmHg and/or a SpO_2_ of <94% despite being on COT. Dogs on COT who demonstrated persistently increased RR and/or effort (in the absence of confounding factors such as pain or stress as determined by the primary clinician) or SpO_2_ of <94% were considered to have failed COT and were candidates for escalation to HFNOT. Dogs treated with HFNOT for HRF after failing COT were considered for enrollment. Dogs were excluded if they were treated with HFNOT for reasons other than HRF (e.g., hypercapnic respiratory failure, pleural space disease, and dyshemoglobinemia), if HFNOT was used palliatively, if HFNOT was discontinued due to owner-related factors such as financial constraints or subjective quality of life concerns, or if patients were weaned from MV to HFNOT. All HFNOT-related treatment decisions were dictated by the on-service emergency and critical care resident or faculty board-certified in small animal emergency and critical care. Final case enrollment and exclusion were facilitated by one author from each hospital (JH, CPN).

Demographic information, including age, breed, sex, and body weight in kilograms, was recorded for each patient. Known or suspected etiology of HRF, as determined by thoracic radiographs or computed tomography, airway sampling (e.g., transtracheal wash and bronchoalveolar lavage), echocardiography, visual airway exam, and/or necropsy, was also recorded. Dogs were considered to have HRF of unknown etiology if a definitive or suspected diagnosis was not identified on imaging, laboratory diagnostics, transtracheal wash or bronchoalveolar lavage sample cytology or culture, and/or necropsy, or if euthanasia or death occurred before a diagnosis could be determined. Dogs were classified as having multiple respiratory diseases if they were diagnosed with two or more respiratory conditions. Additional case information collected included the duration of HFNOT, the duration of hospitalization, Acute Patient Physiologic and Laboratory Evaluation (APPLE_fast_) score (21), a form of COT utilized before HFNOT, whether or not the patient was successfully weaned from HFNOT, and the reason for failure to wean if applicable, and the cause of death or rationale for euthanasia. Dogs were divided into two groups. Dogs successfully weaned from HFNOT to either COT or room air who sustained normal respiratory parameters for over 24 h were categorized into the HFNOT success group. Dogs in the HFNOT failure group were those who were escalated to MV, died on HFNOT due to their respiratory disease, or were euthanized on HFNOT due to lack of response warranting MV and owners’ decisions to discontinue treatment.

HFNOT was delivered using a DRE Volumax VOS Veterinary Oxygen System^1^ or AirvoTM 2 System^2^ at both institutions. HFNOT-specific cannulas, Vapotherm nasal cannula,^3^ and OptiflowTM+,^4^ provided by the manufacturer of DRE Volumax Veterinary Oxygen System (see text footnote 1) or AirvoTM 2 System (see text footnote 2), respectively, were used. Nasal cannula type and size were selected based on patient size and nasal conformation, with the goal of occluding no more than 50% of the nares as recommended by the manufacturers. Patients were closely monitored for HFNOT-associated complications. HFNOT flow rate (L/kg/min) and delivered FiO_2_ were titrated to maintain a SpO_2_ of 94–97%, measured with a Radical-7 pulse oximeter^5^ or MasimoRad-57 pulse oximeter^6^ at both institutions. SpO_2_ readings were considered accurate if there was an appropriate plethysmographic waveform and if the reported HR matched the HR as determined by cardiac auscultation, pulse palpation, or electrocardiography. HFNOT was typically initiated at a flow rate of 1–1.5 L/kg/min and was then adjusted within a range of 0.5–2.5 L/kg/min to balance patient comfort and SpO_2_ optimization. FiO_2_ was initially set at 1.0 and then titrated between 0.21 and 1.0 to maintain target SpO_2_. HFNOT temperature was maintained at 37°C in most cases but was decreased to as low as 33°C in patients experiencing hyperthermia (rectal temperature greater than 39°C) or set at 34°C in patients with premature or neonate nasal cannula on AirvoTM 2 System (see text footnote 2). The HFNOT protocol utilized in this study is provided in Supplementary material 1. Sedation was administered as deemed necessary by the primary clinician to facilitate the application of HFNOT nasal cannulas and/or improve tolerance of HFNOT. Sedation medication, timing of administration, and the route of administration were recorded as applicable.

The following variables, as available, were collected before HFNOT initiation (up to 1 h prior), within 30 min after initiating HFNOT (T0), and at variable time intervals throughout HFNOT, ranging from every hour to every 8 h depending on patient acuity and hospital staffing: RR, HR, SpO_2_, FiO_2_, HFNOT flow rate (L/min), and HFNOT temperature (°C). The measurement of these variables before and throughout HFNOT allowed the calculation of ROX, SF, and ROX-HR.

When possible, PaO_2_ and PaCO_2_ were also measured before HFNOT initiation (up to 1 h prior), within 30 min after starting HFNOT, and at variable intervals during HFNOT (as determined by the primary clinician) via arterial blood gas analysis using a Stat Profile PRIME Plus VET Critical Care blood gas analyzer^7^ at the Ohio State University or IDEXX VetStat analyzer^8^ at Université de Lyon. Arterial blood samples were acquired with intermittent arterial venipuncture or aspiration from an arterial catheter using a standard three-syringe technique (22). APPLE_fast_ scores were calculated based on physical exam parameters obtained upon hospital admission and laboratory diagnostics performed within the first 24 h of hospitalization.

### Statistical analysis

All data analyses were performed using SAS 9.4 Analytics Software.^9^ A significance threshold of *p* < 0.05 was used, and the significance was adjusted using the Benjamini and Hochberg linear step-up false discovery method (23) for multivariate analyses as appropriate. Data was delivered in a Microsoft Excel^10^ spreadsheet when analysis was complete.

Dogs were divided into HFNOT success or failure groups as defined above. Data were evaluated for normality by histogram, skewness, and Q-Q plot evaluation. All demographic and HFNOT outcome predictor parameters were not normally distributed and therefore reported in terms of medians and interquartile ranges (IQR). Age, body weight, APPLE_fast_, and HFNOT treatment duration were compared between HFNOT outcome groups using Mann–Whitney U non-parametric tests, and sex was compared between groups using Fisher’s exact test. Respiratory rate, PaO_2_, PaCO_2_, and SpO_2_ measured within 1 h before and within 30 min after starting HFNOT were compared between outcome groups using Wilcoxon-signed rank tests.

Variables considered as potential HFNOT outcome predictive parameters included RR, ROX, ROX-HR, SpO_2_, FiO_2_, SF, and HFNOT flow rate. Logistic regressions were used to test baseline (T0) predictor values for relationships to odds of success. For each predictor, all measurements obtained between 0 and 16 h of treatment were averaged, and then logistic regressions were run on the averages. The *p*-values were adjusted for multiple comparisons with the linear step-up false discovery method of Benjamini and Hochberg (23). For each predictor with *p* < 0.05, logistic regressions were run separately for each treatment for 1–16 h. Log-likelihood, profile-likelihood confidence intervals, and the area under the receiver operating curve (AUC) were recorded. Confidence intervals (CI) and AUC were recorded for each logistic regression. The predictive value was considered inadequate with an AUC of <0.70, acceptable with an AUC of 0.70–0.80, excellent with an AUC of 0.80–0.90, and outstanding with an AUC of >0.90 (24).

## Results

**Figure 1:**
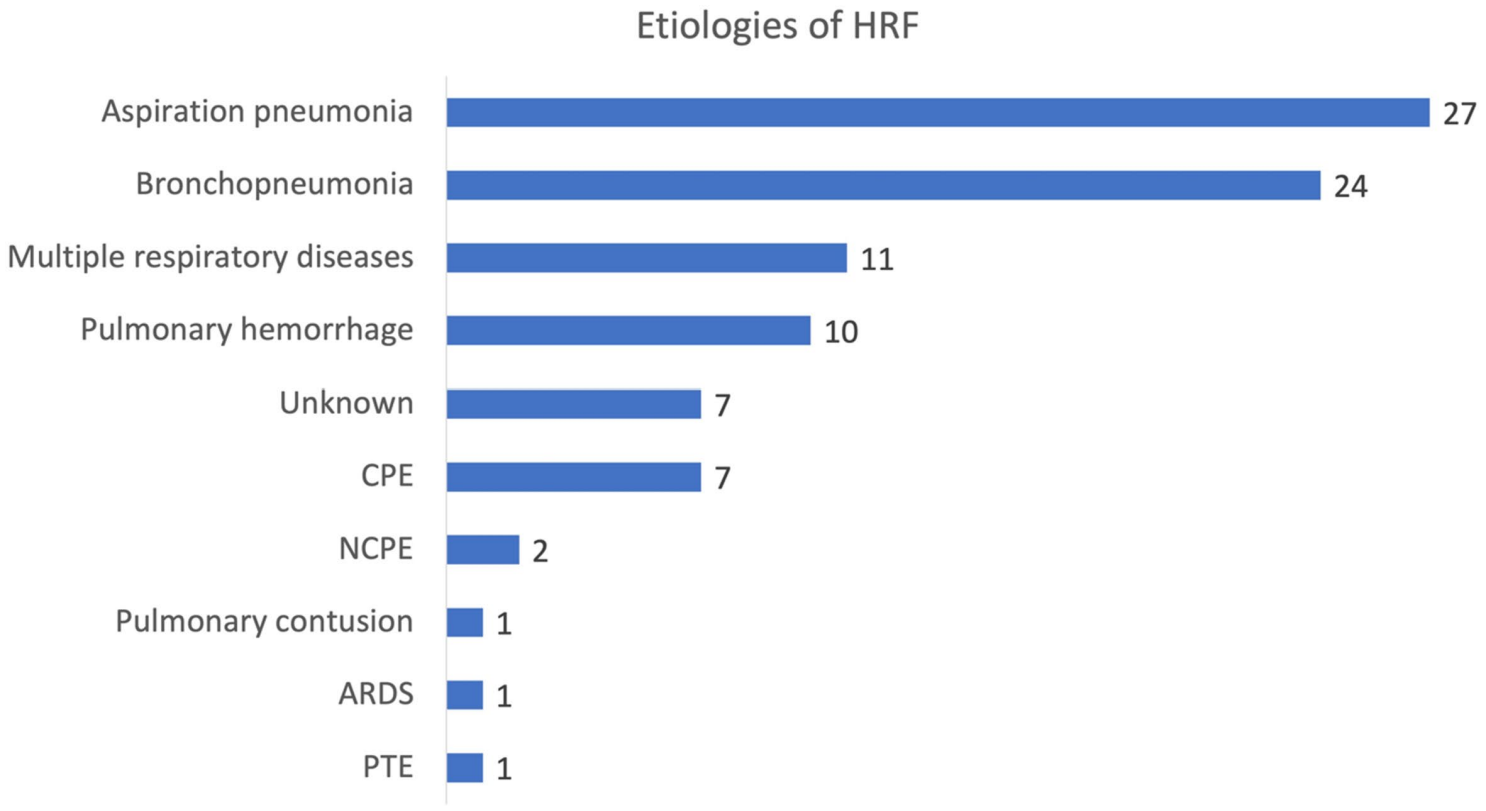
Etiologies of hypoxemic respiratory failure (HRF) treated with high-flow nasal cannula oxygen therapy in this study population. HRF, hypoxemic respiratory failure; CPE, cardiogenic pulmonary edema; NCPE, non-cardiogenic pulmonary edema; ARDS, acute respiratory distress syndrome; PTE, pulmonary thromboembolism.

In total, 88 dogs treated with HFNOT for HRF met the inclusion criteria for enrollment in this cohort. Demographic data describing the included dogs were similar between the HFNOT success and failure groups; these data are displayed in Table 1. Median age, sex, and weight were not significantly different between groups (all *p* < 0.05). The median (IQR) APPLE_fast_ score for the entire cohort was 26 (12–49), and APPLE_fast_ scores did not differ significantly between the success and failure groups (*p* < 0.05). Mixed breed dogs (*N* = 19), Labrador Retrievers (*N* = 7), and French Bulldogs (*N* = 5) were the three most commonly represented breeds. Other represented breeds are provided in Supplementary Table 1. Etiologies of HRF represented in the study population are depicted in Figure 1. Pneumonia was the most common cause of HRF diagnosed in this cohort. In total, 27 of the 51 dogs treated with HFNOT for pneumonia were diagnosed with aspiration pneumonia, while the remainder were diagnosed with various other etiologies of infectious bronchopneumonia, such as canine infectious respiratory disease complex and Blastomycosis. HFNOT duration was the only descriptive data point that differed significantly between groups. HFNOT duration was significantly longer in the success group compared to the failure group (*p <* 0.001), with a median HFNOT duration of 24 h (IQR 10–47 h) in the success group and a median HFNOT duration of 8 h (IQR 4–17 h) in the failure group. No complications associated with HFNOT were observed in any of the dogs.

**Table 1:** Demographic data for 88 dogs treated with high-flow nasal cannula oxygen therapy.

Demographic variables	Success (*N* = 33)	Failure (*N* = 55)	*p*-value
Age (years)	7 (3–12)	8 (2–11)	0.911
Weight (kg)	21 (7–33)	17 (10–29)	1.000
Sex			0.608
Male intact	9	10	
Male neutered	14	24	
Female intact	2	2	
Female spayed	8	19	
APPLE_fast_	24 (19–29)	25 (21–28)	0.5014

Age, sex, and weight are reported as median (interquartile range).

The majority of patients received at least one dose of a sedative medication over the course of their HFNOT treatment period. The most commonly administered sedative medication was butorphanol (0.1–0.3 mg/kg IV every 2–8 h or as a constant rate infusion of 0.1–0.4 mg/kg/h IV). The most commonly employed second-line sedation agents were either acepromazine (0.01–0.03 mg/kg IV every 2–6 h) or dexmedetomidine (0.5–2 mcg/kg IV or 0.5–2 mcg/kg/h as a constant rate infusion IV).

**Figure 2:**
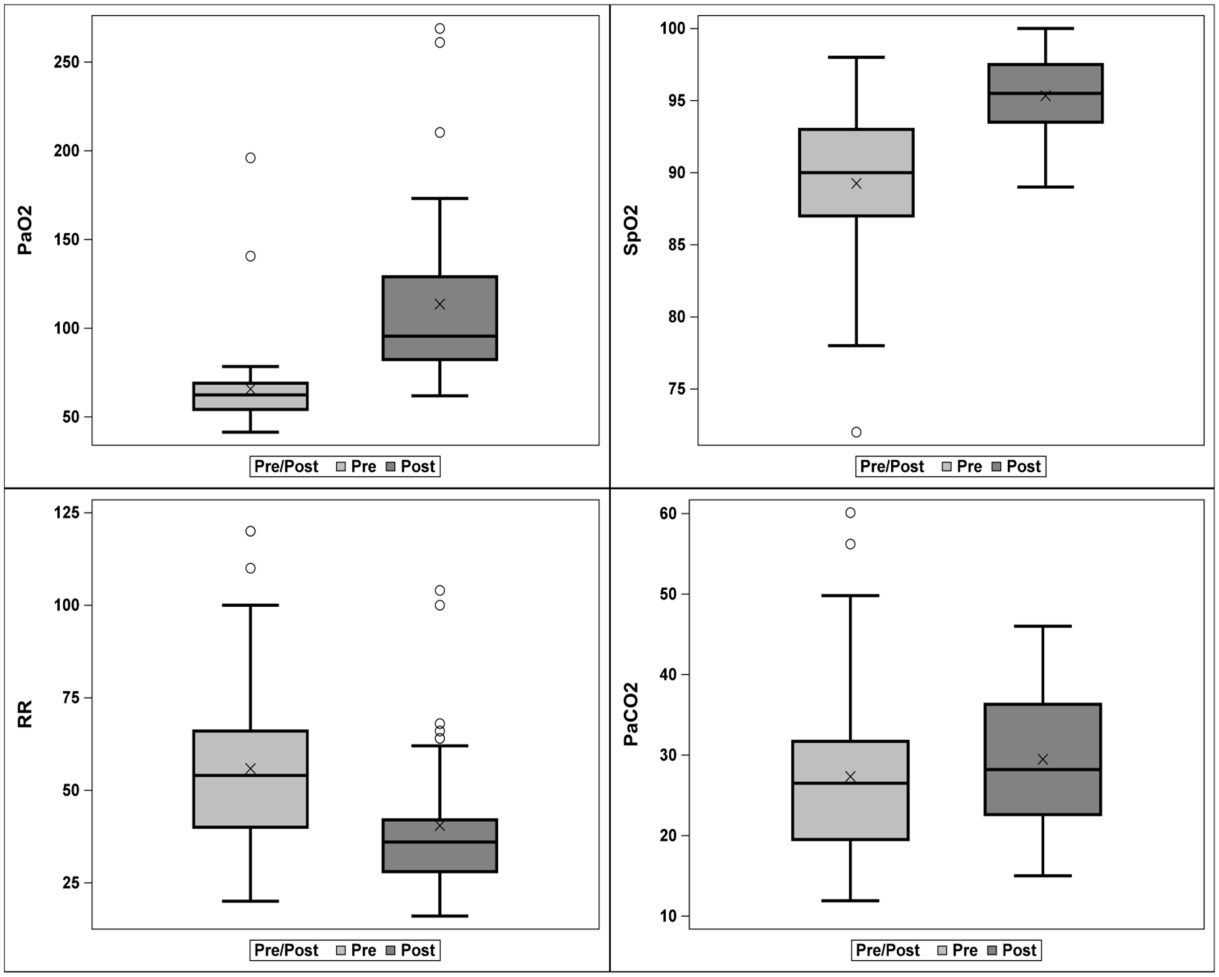
Box and whisker plot of respiratory function indices within 1 h before and within 30 min after starting high-flow nasal cannula oxygen therapy (HFNOT). Each box represents the second and third quartiles, the whiskers delimit the range, the bar in each box represents the first and fourth quartiles, and the circles represent outliers. PaO_2_ and PaCO_2_ are reported in units of mmHg, SpO_2_ in units of percent, and RR in units of breaths per minute. The levels of PaO_2_ and SpO_2_, as well as RR, improved significantly (*p* < 0.001), while the level of PaCO_2_ increased significantly (*p* = 0.006). HFNOT, High flow nasal cannula oxygen therapy; SpO_2_, pulse oximetry saturation; RR, respiratory rate.

The impact of HFNOT on traditional indices of oxygenation and ventilation, including PaO_2_, SpO_2_, PaCO_2_, and RR, are summarized in Figure 2. PaO_2_ (mmHg), SpO_2_ (%), and RR (respiration per minute; rpm) were significantly improved, characterized by increases in PaO_2_ (Pre-HFNOT: *n* = 41, median 62.4 mmHg, IQR 54.2–69.0 mmHg; Post-HFNOT: *n* = 39, median 95.5 mmHg, IQR 82.3–129.0 mmHg) and SpO_2_ (Pre-HFNOT: *n* = 51, median 90%, IQR 87–93%; Post-HFNOT: *n* = 49, 95%, IQR 93–97%) and decrease in RR (Pre-HFNOT: *n* = 46, median 54 rpm, IQR 40–66 rpm; Post-HFNOT: *n* = 43, median 36 rpm, IQR 28–42 rpm) (all *p* < 0.001). PaCO_2_ was mildly increased within 30 min of initiating HFNOT (Pre-HFNOT: *n* = 39, median 27 mmHg, IQR 20–32 mmHg; Post: *n* = 39, median 28 mmHg, IQR 22–36 mmHg; *p* = 0.006).

In total, 33 of 88 dogs (38%) met the inclusion criteria in the HFNOT success group, while 55 of 88 dogs (62%) failed HFNOT. Of the 33 dogs in the HFNOT success group, 25 dogs survived to discharge. A total of 16 out of 25 dogs were weaned to room air, while 9 out of 25 dogs were weaned to COT (oxygen cage or nasal cannulas). Eight dogs in the HFNOT success group (24%) did not survive to discharge: four of these dogs were euthanized due to poor prognosis associated with concurrent neoplasia (heart-based tumor, unspecified metastatic disease, pituitary and hepatic neoplasia, and leukemia, respectively); one dog was euthanized due to worsening cardiac arrhythmias; two dogs were euthanized due to a reportedly poor prognosis from systemic disease unrelated to pulmonary disease; and one dog experienced respiratory arrest secondary to upper airway obstruction. Of those dogs who failed HFNOT, 2 dogs were escalated to MV, 14 dogs died on HFNOT, and 37 dogs were euthanized due to lack of HFNOT response warranting MV. The two dogs escalated to MV and survived to discharge. The overall survival to discharge was 31% (27/88). ROX, ROX-HR, SF, SpO_2_, RR, FiO_2_, and APPLE_fast_ score were assessed for their ability to predict HFNOT outcome at T0 (Table 2). None of the parameters evaluated were predictive of HFNOT outcome when measured at the time of HFNOT initiation (all adjusted *p* > 0.05). These variables were also assessed for their ability to predict HFNOT outcome when averaged over treatment for 0–16 h and at each time point for 1–16 h of treatment. Odds ratios of HFNOT failure and AUC determination for ROX, ROX-HR, SF, SpO_2_, RR, and FiO_2_ when averaged over 0–16 h of the HFNOT treatment are detailed in Table 3. With the exception of RR, all parameters (ROX, ROX-HR, SF, SpO_2_, and FiO_2_) were adequately predictive (all AUC > 0.7, adjusted *p* < 0.05) of HFNOT outcome when averaged over the treatment period. Of the parameters assessed, SF was most strongly predictive of HFNOT outcome, with an AUC of 0.77 (95% CI 0.66–0.87, *p* = 0.002). For every 10-unit increase in median SF over this time period, the odds of HFNOT success increased by 13% (Odds ratio; OR 1.13, 95% CI 1.05–1.23). By comparison, ROX was adequately but less strongly predictive of HFNOT response, with an AUC of 0.72 (95% CI 0.60–0.83, *p* = 0.005). For every 1 unit increase in average ROX, the odds of HFNOT success increased by 26% (OR 1.26, 95% CI 1.09–1.52). The predictive value of ROX-HR was slightly stronger than that of ROX alone, with an AUC of 0.74 (95% CI 0.63–0.85, *p* = 0.002). For every 1 unit increase in ROX-HR, the odds of HFNOT success increased by 26% (OR 1.26, 95% CI 1.09–1.50).

**Table 2:** Odds ratios for high-flow nasal cannula oxygen therapy (HFNOT) failure, *p*-values, and area under the curve (AUC) for each predictive baseline variable collected within 30 min following initiation of HFNOT.

Predictive variables	*N*	OR (95% CI)^a^	*p*-value^b^	Adjusted *p*-value^c^	AUC (95%CI)^d^
ROX	84	1.23 (0.93–1.66)	0.141	0.273	0.62 (0.50–0.75)
ROX-HR	67	1.30 (1.01–1.78)	0.038	0.191	0.72 (0.60–0.85)
SF	84	1.06 (0.98–1.15)	0.156	0.273	0.63 (0.50–0.75)
SpO_2_	84	1.08 (1.00–1.19)	0.055	0.191	0.62 (0.50–0.75)
RR	84	0.94 (0.82–1.07)	0.381	0.445	0.55 (0.42–0.68)
FiO_2_	84	0.88 (0.72–1.07)	0.200	0.280	0.58 (0.46–0.71)
APPLE_fast_	65	1.00 (0.92–1.08)	0.988	0.988	0.56 (0.40–0.71)

^a^Logistic regression. ^b^Log-likelihood *p*-value. ^c^False discovery rate adjusted *p*-value. ^d^Area under the Receiver Operating Characteristics (ROC) Curve.

HFNOT, High flow nasal cannula oxygen therapy; AUC, area under the receiver operating curve; N, number of measured points; OR, odds ratio; CI, confidence interval; ROX, respiratory rate-oxygenation index; ROX-HR, modified ROX index; SF, ratio of pulse oximetry saturation to fraction of inspired oxygen; SpO_2_, pulse oximetry saturation; FiO_2_, fraction of inspired oxygen; APPLE_fast_, Acute Patient Physiologic and Laboratory Evaluation.

**Table 3:** Odds ratios for high-flow nasal cannula oxygen therapy (HFNOT) failure, *p*-values, and area under the curve (AUC) for each median value of a predictive variable over treatment at 0–16 h of HFNOT.

Predictive variables	*N*	OR (95% CI)^a^	*p*-value^b^	Adjusted *p*-value^c^	AUC^d^
ROX	86	1.26 (1.09–1.52)	0.002	0.005*	0.72 (0.60–0.83)
ROX-HR	79	1.26 (1.09–1.50)	0.001	0.002*	0.74 (0.63–0.85)
SF	87	1.13 (1.05–1.23)	0.000	0.002*	0.77 (0.66–0.87)
SpO_2_	87	1.39 (1.16–1.74)	0.000	0.001*	0.72 (0.62–0.83)
RR	86	0.89 (0.75–1.03)	0.136	0.177	0.60 (0.47–0.72)
FiO_2_	87	0.65 (0.50–0.82)	<0.001	0.001*	0.75 (0.64–0.85)

^a^Logistic regression. ^b^Log-likelihood ratio *p*-value. ^c^False discovery rate adjusted *p*-value. ^d^Area under the receiver operating characteristics (ROC) curve. *Adjusted *p* < 0.05.

HFNOT, High flow nasal cannula oxygen therapy; AUC, area under the receiver operating curve; N, number of measured points; OR, odds ratio; CI, confidence interval; ROX, respiratory rate-oxygenation index; ROX-HR, modified ROX index; SF, ratio of pulse oximetry saturation to fraction of inspired oxygen; SpO_2_, pulse oximetry saturation; FiO_2_, fraction of inspired oxygen; APPLE_fast_, Acute Patient Physiologic and Laboratory Evaluation.

**Figure 3:**
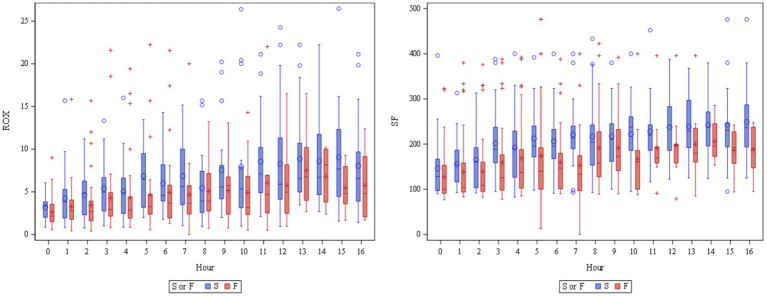
Box and whisker plots of ROX and SF at 0–16 h. While high-flow nasal cannula oxygen therapy (HFNOT) success (S, blue) had significantly higher ROX (adjusted *p* = 0.005) and SF (adjusted *p* = 0.002) when averaged over 0–16 h of treatment compared to HFNOT failure (F, red), this difference was not statistically significant when each time point at 1–16 h was assessed individually. ROX, respiratory-rate oxygenation index; SF, the ratio of pulse oximetry saturation to the fraction of inspired oxygen; HFNOT, high flow nasal cannula oxygen therapy.

ROX, SF, and ROX-HR were then evaluated at each hour within the first 16 h (Supplementary Table 2). When assessed at each hour, there was no time point at which ROX or ROX-HR were adequately predictive (AUC > 0.7) of HFNOT outcome with acceptable significance (*p* < 0.05). SF at 6 h (AUC 0.73, *p* = 0.025) and 7 h (AUC 0.77, *p* = 0.013) were found to be acceptably predictive of HFNOT outcome. The optimal cutoff for predicting HFNC failure at 7 h was SF ≤191 (sensitivity 83% and specificity 76%). ROX and SF values at each 0–16 h compared between HFNOT success and failures are depicted in Figure 3.

## Discussion

The objectives of this study were to prospectively evaluate the utility of ROX, SF, and ROX-HR in predicting HFNOT outcomes for dogs with HRF due to various respiratory diseases and to identify cutoff values for these indices at specific time points during HFNOT. If cutoffs for these predictive indices at specific treatment hours are validated, they could guide timely escalation to MV when necessary. The results of this study indicate that ROX, SF, and ROX-HR were all acceptable predictors (all AUC > 0.7, *p* < 0.05) of HFNOT outcomes, consistent with the findings in human literature and the retrospective study conducted in dogs (1, 4, 17, 18, 20). Furthermore, SF demonstrated acceptable predictive value at a specific hour of HFNOT treatment in this cohort. Nevertheless, our findings demonstrated the predictive utility of all three of these indices in dogs undergoing HFNOT and highlighted the opportunities for further investigation in future studies.

The identification of predictive factors has the potential to improve monitoring of patients on HFNOT to avoid delayed escalation to MV (1, 17). Roca et al. demonstrated the utility of ROX for the first time in 2016, showing it to be a reliable predictor of HFNOT success in human patients with pneumonia-related respiratory failure (1). The study reported that a ROX greater than or equal to 4.88, measured at 2, 6, or 12 h, was consistently associated with a lower risk of escalation to MV. These investigators further validated the diagnostic accuracy of ROX in 2019, demonstrating that an ROX less than 2.85, 3.47, and 3.85 at 2, 6, and 12 h of HFNOT initiation, respectively, were predictive of HFNOT failure (17). Since then, the cutoff value of the ROX for predicting HFNOT outcomes at different time points has continued to be investigated in humans with different etiologies of HRF (4). Following the validation of ROX, multiple variations of ROX have been proposed to further improve the predictive value of the index by incorporating additional parameters. ROX-HR was first suggested by Goh et al. (18). Further studies demonstrated that ROX-HR is a slightly better predictor of HFNOT outcomes compared to ROX in humans with acute HRF (19, 25). Based on these findings, the utility of ROX and ROX-HR as an adjunct monitoring tool for clinicians to manage dogs on HFNOT warrants validation.

The present study represents the first prospective evaluation of outcome predictors in dogs on HFNOT. Despite existing evidence that suggests ROX and ROX-HR are promising tools for predicting HFNOT outcomes in humans, only one retrospective study has explored their utility in dogs that underwent HFNOT for various respiratory diseases (20). The retrospective study revealed that ROX, ROX-HR, and SF were all excellent discriminators of HFNOT success or failure when averaged over the first 15 h of the HFNOT treatment period, with AUC values of 0.75, 0.73, and 0.81, respectively (*p* < 0.05). In addition, ROX and SF showed excellent discriminatory power in predicting HFNOT failure at 6 h, with AUC values of 0.85 (95% CI 0.72–0.99, *p* < 0.002) and 0.87 (95% CI 0.73–0.99, *p* < 0.001), respectively. While the retrospective study yielded strong results, it nonetheless had its limitations. The intervals for measurement of variables necessary for ROX calculation (SpO_2_, RR, and FiO_2_) were not standardized, resulting in a variable number of dogs at each evaluation point for statistical analysis. To address this, our prospective study aimed to collect these variables hourly, resulting in a greater availability of variables at each evaluation point. Moreover, one designated investigator from each participating hospital (JH, CPN) oversaw case enrollment and exclusion in the present study to minimize confounding factors affecting outcome measures, such as euthanasia, due to financial constraints. Finally, both participating institutions employed the same HFNOT protocol to minimize its influence on patient response.

Overall, the findings from the prospective study are consistent with those of the retrospective study. ROX, SF, and ROX-HR were identified as reliable predictors of HFNOT outcome. However, when the predictive values of these indices were averaged over all time points, their predictive power was lower in the prospective study compared to the retrospective cohort despite implementing an enhanced design. Specifically, ROX, SF, and ROX-HR exhibited slightly diminished predictive power, with AUC values ranging from 0.72 to 0.77, contrasting with the retrospective study where AUC values ranged between 0.73 and 0.81. Additionally, when these indices were assessed hourly within the first 16 h, only SF at 6 and 7 h were predictive of HFNOT outcome, in contrast to the retrospective study that identified that SF ratio and ROX had excellent discriminatory power in predicting HFNC outcome. Nevertheless, SF exhibited the highest predictive power at 6 and 7 h, similar to the retrospective study suggesting that 6 h was the most predictive of HFNOT outcome. Based on these findings, the authors suggest that clinicians managing dogs on HFNOT evaluate these variables around 6–7 h following HFNOT initiation to help inform ongoing oxygen support for these patients.

Several potential explanations could account for the overall diminished predictive power of these indices observed in this prospective study. First, disparities in the etiology and severity of HRF between the retrospective and prospective cohorts could have affected the HFNOT outcomes and the performance of predictive variables in these studies. Unlike previous veterinary studies, which reported mortality rates ranging from 45 to 50% in dogs on HFNOT (12–15), 65% of the dogs in the present cohort did not survive to discharge. Interestingly, the predicted mortality rate for this cohort based on the median (IQR) APPLE_fast_ score was only 26% (12–49%), using the equation published by Hayes et al. (21) This predicted mortality is significantly lower than the 65% mortality rate observed in this study. Future studies on the use of HFNOT in dogs may benefit from using the APPLE_full_ score, which might provide a more accurate assessment of overall illness severity and mortality prediction by including both SpO_2_ and RR as indices of respiratory illness instead of relying solely on RR as the APPLE_fast_ score.

Second, the etiologies of HRF represented in the prospective cohort compared to the retrospective cohort may have differed and impacted the performance of these indices. It is important to note that most human studies on the utility of ROX have focused on patients with a homogeneous disease process, such as pneumonia-related hypoxemic failure, pneumonia due to COVID-19, and those who are immunocompromised (3, 4, 9, 26, 27). The fact that the indices showed acceptable predictive utility in this cohort, despite the heterogeneous population of dogs with various disease processes, speaks to the potential for widespread clinical application of these indices across a wide variety of canine patients experiencing HRF. However, further investigation of predictive variables in a more homogeneous population, such as those with aspiration pneumonia, is warranted to determine cutoff values for specific disease processes and more closely mirror human studies of these indices.

Third, the use of sedation in this cohort and its effect on HR, RR, and SpO_2_ might have influenced the predictive value of ROX and ROX-HR in the current study. Sedation has the potential to blunt the impact of sympathetic tone and hypoxemia on increasing HR and RR, potentially resulting in falsely increased ROX and ROX-HR. Sedation can have a variable impact on the accuracy of SpO_2_. Certain vasoconstrictive medications (e.g., dexmedetomidine) reduce pulse oximetry signal strength and increase the reading failure rate (28). Conversely, sedation can reduce motion artifact that also has the potential to reduce SpO_2_ accuracy (29). Sedatives were more frequently administered as intravenous boluses rather than constant rate infusions in this population, so the impact of sedation on the measurement of these indices is likely overshadowed by the large number of data points in this cohort that were not impacted by sedation. Finally, given that this study aimed to assess the utility of these indices in a clinical setting, the type, dose, and frequency of sedative administration were not standardized, thus allowing clinicians autonomy in treatment decisions and making the results more broadly applicable to clinical populations of dogs receiving HFNOT. Future studies that standardize sedation administration could lead to a more precise determination of these indices’ predictive value in dogs and elucidation of the impact of sedation on these indices.

Finally, although efforts were made to minimize confounding factors affecting outcome measures, 67% (37/55) of HFNOT failures resulted in euthanasia. It remains unclear whether dogs that were euthanized while on HFNOT could have been weaned off HFNOT or would still have failed with continued HFNOT. Further research, excluding all euthanized dogs and focusing specifically on dogs that failed HFNOT and were escalated to MV, is necessary.

To the best of the authors’ knowledge, this study represents the largest prospective investigation to date assessing the efficacy of HFNOT in dogs, in addition to being the first prospective study aimed at evaluating predictors of HFNOT outcomes in dogs. The strength of our study lies in its inclusion of the largest number of hypoxemic dogs in a clinical setting to date, reinforcing the results observed in previous literature. Previous research has demonstrated the ability of HFNOT to improve oxygenation parameters in dogs with respiratory failure (12–15). In agreement with the findings in the previous veterinary literature, this study further confirms the efficacy of HFNOT in enhancing oxygenation parameters. Dogs failing to respond to COT exhibited a significant increase in PaO_2_ and SpO_2_ and a decrease in RR within 30 min of HFNOT initiation compared to values obtained within the preceding hour before HFNOT commencement (Figure 2). Overall, this study reinforces the efficacy of HFNOT in dogs failing COT.

Interestingly, a very mild but statistically significant increase in PaCO_2_ following the initiation of HFNOT in dogs compared to the pre-treatment levels was identified in the present cohort (Figure 2). Research on HFNOT and its impact on PaCO_2_ levels has shown varied outcomes in both healthy sedated dogs and those with hypoxemia (10–14, 30). For instance, Jagodich and colleagues observed a significant increase in PaCO_2_ in eight healthy sedated dogs post-HFNOT (10). In contrast, Daly and colleagues demonstrated a trend of increasing PaCO_2_ with higher flow rates in a cohort of six healthy, sedated dogs undergoing HFNOT, but this difference was not found to be statistically significant (30). In hypoxemic dogs, Keir and colleagues noted a slight yet significant rise in PaCO_2_ post-HFNOT, which did not lead to significant respiratory acidosis (14). Conversely, the HOT-DOG study did not report any significant PaCO_2_ changes despite noting a trend of moderate positive correlation between HFNOT flow rate and PaCO_2_ (13). Notably, moderate to severe hypercapnia (PaCO_2_ > 50 mm Hg) was recorded in three out of five brachycephalic dogs recovering from anesthesia under HFNOT (11). The cause of this observation is likely multifactorial. Previous studies suggested that this observed PaCO_2_ increase may result from sedation-induced decreased RR, heightened exhalation resistance due to high gas flow rates, or underlying pulmonary conditions. It is important to note that in our study, PaCO_2_ levels before HFNOT were abnormally low due to hyperventilation from severe hypoxemia. As HFNOT effectively raises PaO_2_, it reduces the hypoxemic respiratory drive, potentially explaining the slight increase in PaCO_2_. While the difference in PaCO_2_ pre- and post-HFNOT was statistically significant in this study, the median pre- and post-HFNOT PaCO_2_ values were both below the normal reference range of 30–40 mmHg, calling into question the clinical significance of this finding, particularly in the absence of an associated respiratory alkalosis. Future studies are warranted to investigate the impact of HFNOT on PaCO_2_ and its clinical significance in dogs.

This study has some limitations. Considering the observational nature of this investigation, all aspects of case management were at the discretion of the primary clinician. Differences in patient temperament, the frequency of sedation administration, and triggers and timing of recommendation for escalation to MV may have contributed to the reduced predictive power of ROX and ROX-HR in patients who were more frequently and heavily sedated. Inherent limitations of SpO_2_, such as motion artifacts, reduced accuracy due to peripheral vasoconstriction from dexmedetomidine, varied interpersonal interpretations, and mucous membrane pigmentation, could have impacted the performance of the SF, ROX, and ROX-HR indices. Future prospective studies validating the utility of ROX, SF, and ROX-HR as indices of HFNOT outcome may benefit from the standardization of sedation protocols, monitoring frequency, and triggers for the recommendations for MV.

## Conclusion

In this large population of dogs with HRF treated with HFNOT, ROX, SF, and ROX-HR were acceptable predictors of HFNOT outcome. These indices were easily obtained and have the potential to predict HFNOT response that can help guide clinician’s decisions to escalate or de-escalate oxygen support in the future. Additionally, consistent with previous research in dogs, HFNOT demonstrated effective enhancement of oxygenation parameters. This study serves as a stepping stone for future investigations aimed at validating the predictive utility of ROX, SF, and ROX-HR in a specific disease population and assessing the predictive value of these indices in a population of dogs that fail HFNOT and subsequently are escalated to MV.

## Data availability statement

The raw data supporting the conclusions of this article will be made available by the authors, without undue reservation.

## Ethics statement

The animal studies were approved by The Ohio State University, Université de Lyon. The studies were conducted in accordance with the local legislation and institutional requirements. Written informed consent was obtained from the owners for the participation of their animals in this study.

## Author contributions

ED: Investigation, Validation, Writing – original draft, Writing – review & editing, Formal analysis. JH: Formal analysis, Investigation, Validation, Writing – original draft, Writing – review & editing, Conceptualization, Data curation, Funding acquisition, Methodology, Project administration, Resources, Software, Supervision. IP: Investigation, Validation, Writing – original draft, Writing – review & editing. JL: Investigation, Writing – original draft, Writing – review & editing. BA: Conceptualization, Data curation, Investigation, Writing – original draft, Writing – review & editing. CP-N: Conceptualization, Data curation, Investigation, Methodology, Supervision, Validation, Writing – original draft, Writing – review & editing.

## Footnotes

^1^DRE Volumax VOS Veterinary Oxygen System, Avante, Louisville, KY.

^2^Fisher & Paykel AirvoTM 2 System, Fisher & Paykel Healthcare, Panmure, Aukland, New Zealand.

^3^High-flow therapy nasal cannula, Vapotherm Inc., Exeter, NH.

^4^OptiflowTM+ nasal high-flow cannula, Fisher & Paykel Healthcare, Panmure, Aukland, New Zealand.

^5^Radical-7 pulse oximeter, Masimo Corporation, Irvine, CA.

^6^MasimoRad-57 pulse oximeter, Masimo Corporation, Irvine, CA.

^7^Stat Profile PRIME Plus VET Critical Car blood gas analyzer, Nova Biomedical, Billerica, MA.

^8^IDEXX Laboratories, Westbrook, ME.

^9^SAS 9.4 Analytics Software, SAS Institute, Cary, NC.

^10^Excel, Microsoft 365, Microsoft Corporation, Redmond, WA.
