# Supplementary material for: The utility of the respiratory rate-oxygenation index as a predictor of treatment response in dogs receiving high-flow nasal cannula oxygen therapy
Source: Front Vet Sci. 2024 May 7;11:1404195. doi: 10.3389/fvets.2024.1404195 (PMC11106722; doi:10.3389/fvets.2024.1404195)
Supplement: Supplementary file 1 [file Data_Sheet_1.docx]

Supplemental Material 1. High-flow nasal cannula oxygen therapy protocol utilized in this study.

High-flow titration protocol

**Targets**

1. SpO2 of 94-97% or PaO2 of 80-100 mm Hg
2. Respiratory rate (< 60 if not anxious) and normal effort

- Machine setting at initiation
  - Flow rate: _____ L/min (In general, 1-1.5 L/kg/min; min 0.5, max 2.5 L/kg/min)
  - FiO2: _____ % (60-100% and titrate down to 60% or lower after 15-30 minutes from initiation as long as target is maintained)
  - Temperature 37 C (Can decrease down to 33 C depending on patient’s RR/RE)

High-flow escalation protocol

- If SpO2 < 94% (PaO2< 80) less than target, then titrate up FiO2 and flow rate **every 10-15** in **alternating fashion** until target achieved or max reached:
  - Increase FiO2 by _____ (typically 5-10 %), max _____%
  - Increase flow rate by _____ L/min (typically 1-10 L/min), max _____ L/min
  - Alert clinician if target not achieved despite max flow rate and FiO2
- If concern for acute decompensation (insufficient time for escalation), place patient on initiation settings and contact clinician

High-flow de-escalation protocol

- Initiate if target SpO2 or PaO2 is achieved for ____ hours
  - Decrease FiO2 by _____ % every _____ minutes until it reaches 30-40%
- And then
  - Decrease Flow rate by ____ L/min (1-10 L/min) every ____ minutes until 0.5 L/kg/min and then discontinue
