# Supplementary material for: The utility of the respiratory rate-oxygenation index as a predictor of treatment response in dogs receiving high-flow nasal cannula oxygen therapy
Source: Front Vet Sci. 2024 May 7;11:1404195. doi: 10.3389/fvets.2024.1404195 (PMC11106722; doi:10.3389/fvets.2024.1404195)
Supplement: Supplementary file 2 [file Table_1.docx]

Supplemental Table 1. Breeds represented in this cohort.

| **Breeds** | **Total** | **Failure** | **Success** |
| --- | --- | --- | --- |
| Mixed Breed | 19 | 13 | 6 |
| Labrador Retriever | 7 | 5 | 2 |
| French Bulldog | 5 | 3 | 2 |
| Great Dane | 4 | 3 | 1 |
| Cavalier King Charles Spaniel | 4 | 2 | 2 |
| German Shepherd | 3 | - | 3 |
| Bulldog | 3 | 2 | 1 |
| Australian Shepherd | 3 | 3 | - |
| Weimaraner | 3 | 1 | 2 |
| English Bulldog | 2 | 1 | 1 |
| Rottweiler | 2 | - | 2 |
| American Bulldog | 2 | - | 2 |
| Dachshund | 2 | 2 | - |
| Beagle | 2 | 1 | 1 |
| West Highland White Terrier | 2 | 2 | - |
| Jack Russel Terrier | 2 | 1 | 1 |
| Golden Retriever | 2 | 2 | - |
| Standard Poodle | 1 | 1 | - |
| Cane Corso | 1 | - | 1 |
| Neapolitan Mastiff | 1 | 1 | - |
| Pekingese | 1 | 1 | - |
| Scottish Terrier | 1 | 1 | - |
| Japanese Chin | 1 | 1 | - |
| Boxer | 1 | - | 1 |
| St. Bernard | 1 | 1 | - |
| Wheaten Terrier | 1 | - | 1 |
| Staffordshire Terrier | 1 | 1 | - |
| Irish Wolfhound | 1 | - | 1 |
| Cocker Spaniel | 1 | - | 1 |
| Coton de Tulear | 1 | 1 | - |
| Spitz | 1 | - | 1 |
| Yorkshire Terrier | 1 | 1 | - |
| Husky | 1 | 1 |  |
| Chinese Crested | 1 | 1 | - |
| Hovawart | 1 | 1 | - |
| Bichon Frise | 1 | - | 1 |
| Doberman | 1 | - | 1 |
| Maltese | 1 | - | 1 |

Supplemental Table 2. Odds ratios for high-flow nasal cannula oxygen therapy (HFNOT) failure, *P* values, and Area Under the Curve (AUC) for ROX, ROX-HR, and SF at each treatment hour 1-16.

| **Predictive variables** | **Treatment Hour** | **N** | **OR (95% CI)** | ***P* value^b^** | **AUC^c^** |
| --- | --- | --- | --- | --- | --- |
| ROX | 1 | 68 | 1.14 (0.95-1.42) | 0.155 | 0.61 (0.47-0.76) |
|  | 2 | 78 | 1.15 (0.98-1.38) | 0.079 | 0.66 (0.54-0.79) |
|  | 3 | 52 | 1.07 (0.94-1.26) | 0.317 | 0.68 (0.53-0.83) |
|  | 4 | 64 | 1.04 (0.92-1.19) | 0.521 | 0.63 (0.48-0.77) |
|  | 5 | 46 | 1.13 (0.98-1.33) | 0.087 | 0.72 (0.56-0.87) |
|  | 6 | 53 | 1.06 (0.93-1.23) | 0.360 | 0.66 (0.51-0.81) |
|  | 7 | 39 | 1.14 (0.98-1.38) | 0.102 | 0.68 (0.51-0.86) |
|  | 8 | 40 | 1.03 (0.86-1.23) | 0.762 | 0.49 (0.3-0.67) |
|  | 9 | 33 | 1.16 (0.97-1.5) | 0.103 | 0.61 (0.4-0.81) |
|  | 10 | 31 | 1.11 (0.97-1.35) | 0.137 | 0.66 (0.46-0.86) |
|  | 11 | 27 | 1.1 (0.94-1.36) | 0.244 | 0.69 (0.46-0.91) |
|  | 12 | 30 | 1.09 (0.95-1.31) | 0.239 | 0.59 (0.37-0.81) |
|  | 13 | 26 | 1.05 (0.91-1.26) | 0.524 | 0.55 (0.3-0.8) |
|  | 14 | 23 | 1.09 (0.90-1.43) | 0.385 | 0.54 (0.26-0.82) |
|  | 15 | 25 | 1.21 (0.98-1.64) | 0.080 | 0.68 (0.47-0.9) |
|  | 16 | 24 | 1.10 (0.93-1.38) | 0.304 | 0.61 (0.35-0.87) |
| ROX-HR | 1 | 47 | 1.15 (1.00-1.45) | 0.056 | 0.67 (0.49-0.85) |
|  | 2 | 56 | 1.12 (1.01-1.32) | 0.026 | 0.69 (0.54-0.85) |
|  | 3 | 32 | 1.06 (0.88-1.29) | 0.534 | 0.68 (0.48-0.88) |
|  | 4 | 47 | 1.13 (1.00-1.31) | 0.053 | 0.66 (0.49-0.84) |
|  | 5 | 26 | 1.15 (0.95-1.44) | 0.148 | 0.72 (0.49-0.94) |
|  | 6 | 35 | 1.06 (0.87-1.28) | 0.540 | 0.64 (0.44-0.84) |
|  | 7 | 24 | 1.22 (0.98-1.68) | 0.080 | 0.81 (0.63-1) |
|  | 8 | 31 | 1.10 (0.92-1.38) | 0.299 | 0.6 (0.39-0.8) |
|  | 9 | 19 | 1.17 (0.91-1.60) | 0.229 | 0.64 (0.37-0.92) |
|  | 10 | 16 | 1.09 (0.94-1.34) | 0.272 | 0.66 (0.37-0.94) |
|  | 11 | 14 | 1.04 (0.90-1.23) | 0.607 | 0.69 (0.38-1) |
|  | 12 | 18 | 1.02 (0.88-1.19) | 0.811 | 0.57 (0.24-0.9) |
|  | 13 | 16 | 1.01 (0.86-1.18) | 0.934 | 0.57 (0.25-0.89) |
|  | 14 | 12 | 1.11 (0.89-1.61) | 0.382 | 0.63 (0.2-1) |
|  | 15 | 14 | 1.21 (0.97-1.90) | 0.104 | 0.76 (0.5-1) |
|  | 16 | 14 | 1.08 (0.85-1.47) | 0.545 | 0.58 (0.25-0.9) |
| SF | 1 | 69 | 1.04 (0.96-1.12) | 0.313 | 0.69 (0.56-0.81) |
|  | 2 | 78 | 1.06 (0.99-1.15) | 0.090 | 0.68 (0.56-0.8) |
|  | 3 | 52 | 1.06 (0.99-1.15) | 0.071 | 0.72 (0.58-0.87) |
|  | 4 | 65 | 1.04 (0.98-1.11) | 0.241 | 0.64 (0.51-0.78) |
|  | 5 | 47 | 1.05 (0.98-1.12) | 0.160 | 0.7 (0.55-0.86) |
|  | **6** | **53** | **1.09 (1.01-1.20)** | **0.025** | **0.73 (0.58-0.88)*** |
|  | **7** | **39** | **1.11 (1.02-1.23)** | **0.013** | **0.77 (0.61-0.93)*** |
|  | 8 | 40 | 1.03 (0.96-1.10) | 0.398 | 0.6 (0.41-0.78) |
|  | 9 | 33 | 1.04 (0.96-1.15) | 0.338 | 0.62 (0.42-0.82) |
|  | 10 | 31 | 1.10 (1.00-1.24) | 0.054 | 0.76 (0.57-0.94) |
|  | 11 | 27 | 1.07 (0.96-1.23) | 0.231 | 0.71 (0.49-0.93) |
|  | 12 | 30 | 1.08 (0.97-1.24) | 0.151 | 0.66 (0.44-0.87) |
|  | 13 | 26 | 1.08 (0.97-1.25) | 0.185 | 0.69 (0.46-0.92) |
|  | 14 | 24 | 1.11 (0.95-1.34) | 0.191 | 0.66 (0.41-0.9) |
|  | 15 | 25 | 1.13 (0.99-1.36) | 0.073 | 0.71 (0.5-0.92) |
|  | 16 | 24 | 1.15 (1.00-1.40) | 0.050 | 0.69 (0.46-0.92) |

^a^ Logistic regression, profile-likelihood confidence intervals

^b^ Log-likelihood *P* value

^c^ Area under the receiver operating characteristic (ROC) curve

^*^ Denotes significance with a *P* value < 0.05 and an AUC > 0.70

Abbreviations – High flow nasal cannula oxygen therapy, HFNOT; area under the receiver operating curve, AUC; confidence interval, CI; modified ROX index, ROX-HR; N, number of measured points; odds ratio, OR; respiratory rate-oxygenation index, ROX; ratio of pulse oximetry to fraction of inspired oxygen, SF; respiratory rate, RR
